# Co-expression of vascular endothelial growth factor (VEGF) and its receptors (flk-1 and flt-1) in hormone-induced mammary cancer in the Noble rat

**DOI:** 10.1038/sj.bjc.6692206

**Published:** 1999-12

**Authors:** B Xie, N N C Tam, S W Tsao, Y C Wong

**Affiliations:** Department of Anatomy, Faculty of Medicine, The University of Hong Kong, Li Shu Fan Building, 5 Sassoon Road, Hong Kong

**Keywords:** mammary cancer, angiogenesis, VEGF, VEGF receptors, autocrine regulation

## Abstract

Vascular endothelial growth factor (VEGF) is recognized to play a predominant role in breast cancer prognosis. The action of VEGF is mediated by two high-affinity receptors with ligand-stimulated tyrosine kinase activity: VEGFR-1/flt-1 and VEGFR-2/flk-1, which are expressed mainly in vascular endothelial cells. To the best of our knowledge, no previous studies on the expression of these receptors in breast cancer cells has been made. We have established a new animal model for breast cancer, using a combination of 17β-oestradiol and testosterone as ‘carcinogens’. Taking advantage of the animal model, we have demonstrated that mammary cancer cells expressed not only high levels of VEGF but also, surprisingly, its receptors (flt-1 and flk-1) in mammary cancer cells. Intense reactivities to VEGF, flt-1 and flk-1 were observed in mammary cancer cells, especially in invasive mammary carcinoma. Western blot analysis confirmed the increase in flk-1 and flt-1 proteins in induced mammary cancers. Based on these observations, we hypothesize that in mammary cancer, VEGF regulates, in addition to endothelial proliferation and angiogenesis, also growth of cancer cells by an autocrine mechanism mediated through its receptors. To further verify this hypothesis, we investigated the correlation between cellular proliferation and the expression of VEGF, flt-1 and flk-1. Using double-labelling immunocytochemistry, we have shown a correlation between high VEGF activity and Ki-67 expression. The Ki-67 indices in the areas of strong and weak VEGF reactivities were 58.3% and 3.7% respectively. Similarly, there was also a correlation of strong flk-1 and Ki-67 reactivity. The Ki-67 indices for areas of strong and weak flk-1 reactivities were 53.9% and 3.1% respectively. On the other hand, there was a reverse correlation between flt-1 and Ki-67 activities. These results indicate that overexpression of VEGF and flk-1 is correlated with high Ki-67 index. The data, therefore, suggest that VEGF may act as an autocrine growth factor for mammary cancer cells in vivo and this autocrine regulatory role may be mediated through flk-1. The present study is the first report showing that VEGF may act as a growth stimulator for mammary cancer cells. © 1999 Cancer Research Campaign


*British Journal of Cancer* (1999) **81**(8), 1335–1343
© 1999 Cancer Research Campaign
Article no. bjoc.1999.0849

### Co-expression of vascular endothelial growth factor (VEGF) and its receptors (flk-1 and flt-1) in hormone-induced mammary cancer in the Noble rat

**B Xie, NNC Tam, SW Tsao and YC Wong**

Department of Anatomy, Faculty of Medicine, The University of Hong Kong, Li Shu Fan Building, 5 Sassoon Road, Hong Kong

**Summary** Vascular endothelial growth factor (VEGF) is recognized to play a predominant role in breast cancer prognosis. The action of VEGF is mediated by two high-affinity receptors with ligand-stimulated tyrosine kinase activity: VEGFR-1/flt-1 and VEGFR-2/flk-1, which are expressed mainly in vascular endothelial cells. To the best of our knowledge, no previous studies on the expression of these receptors in breast cancer cells has been made. We have established a new animal model for breast cancer, using a combination of 17β-oestradiol and testosterone as 'carcinogens'. Taking advantage of the animal model, we have demonstrated that mammary cancer cells expressed not only high levels of VEGF but also, surprisingly, its receptors (flt-1 and flk-1) in mammary cancer cells. Intense reactivities to VEGF, flt-1 and flk-1 were observed in mammary cancer cells, especially in invasive mammary carcinoma. Western blot analysis confirmed the increase in flk-1 and flt-1 proteins in induced mammary cancers. Based on these observations, we hypothesize that in mammary cancer, VEGF regulates, in addition to endothelial proliferation and angiogenesis, also growth of cancer cells by an autocrine mechanism mediated through its receptors. To further verify this hypothesis, we investigated the correlation between cellular proliferation and the expression of VEGF, flt-1 and flk-1. Using double-labelling immunocytochemistry, we have shown a correlation between high VEGF activity and Ki-67 expression. The Ki-67 indices in the areas of strong and weak VEGF reactivities were 58.3% and 3.7% respectively. Similarly, there was also a correlation of strong flk-1 and Ki-67 reactivity. The Ki-67 indices for areas of strong and weak flk-1 reactivities were 53.9% and 3.1% respectively. On the other hand, there was a reverse correlation between flt-1 and Ki-67 activities. These results indicate that overexpression of VEGF and flk-1 is correlated with high Ki-67 index. The data, therefore, suggest that VEGF may act as an autocrine growth factor for mammary cancer cells in vivo and this autocrine regulatory role may be mediated through flk-1. The present study is the first report showing that VEGF may act as a growth stimulator for mammary cancer cells. © 1999 Cancer Research Campaign

**Keywords:** mammary cancer; angiogenesis; VEGF; VEGF receptors; autocrine regulation

Breast cancer is the most common cancer and the second most frequent cause of cancer death among women in modern society (Parker et al, 1997). Among all factors, vascular endothelial growth factor (VEGF) is recognized to play a predominant role in breast cancer prognosis (Ref et al, 1997), especially in node-negative breast cancer (Gasparini et al, 1997). VEGF is a glycosylated, multifunctional cytokine that is abundantly expressed and secreted by most human and animal tumours examined thus far (Dvorak et al, 1995). To date, in vivo as well as in vitro experimental studies have demonstrated that VEGF exerts a number of important biological actions on endothelial cells:

1. It acts with a potency some 50 000 times that of histamine to increase the permeability of microvessels to circulating macromolecules (Senger et al, 1983), and this activity is likely to account for the well-documented hyperpermeability of tumour blood vessels (Senger et al, 1993)
2. It is a selective endothelial cell mitogen (Ferrara et al, 1991).

VEGF activity is mediated by two high affinity receptors with ligand-stimulated tyrosine kinase activity: VEGFR-1/flt-1 and VEGFR-2/flk-1 (Hanahan, 1997). Targeted homozygous null mutations of VEGFR-1/flt-1 are essential for endothelial organization during vascular development, while VEGFR-2/flk-1 is required for the formation of blood islands and also for haematopoiesis (Millauer et al, 1993). The expression of these receptors occurs mainly in vascular endothelial cells. This leads most studies to focus on one aspect of the roles of VEGF, i.e. angiogenesis, as a paracrine growth factor for endothelial cells. It is now well recognized that the expression of VEGF is closely related to poor prognosis in breast cancer, despite the fact that the relationship between angiogenesis and poor prognosis remains equivocal (Martin et al, 1997). A number of explanations have been employed for these discrepancies, although none of them are conclusive (Fox and Harris, 1997). In this connection, we hypothesize that VEGF may have other regulatory roles during the development and progression of breast cancer apart from angiogenesis.

More recently, the expression of VEGF receptors has been detected in several types of non-endothelial cells, such as melanoma cell lines (Gitay-Goren et al, 1993), some leukaemic cell lines (Shibuya et al, 1995), NIH 3T3 cells, HeLa cells, Balb/c 3T3 cells (Enomoto et al, 1994), and retinal progenitor cells (Yang and Cepko, 1996), suggesting that VEGF receptors are not restricted to endothelial cells. Moreover, Mascood et al (1997) have reported that in AIDS-associated Kaposi's sarcoma the tumour cells express not only high levels of VEGF but also its

*Received 30 November 1998*
*Revised 2 June 1999*
*Accepted 8 June 1999*

*Correspondence to:* YC Wong receptors. These studies strongly suggest that VEGF may act as an autocrine growth factor both in in vitro and in vivo conditions. To date, however, no study has been made to ascertain whether VEGF also play an autocrine growth regulatory role in adenocarcinoma.

We have established a new animal model for breast cancer, using a combination of 17β-oestradiol and testosterone as 'carcinogens' (Wong et al, 1999; Xie et al, 1999*a*, 1999*b*). In this report, taking advantage of the animal model, we have demonstrated that both the VEGF and its receptors (flt-1 and flk-1) are overexpressed in this hormone-induced mammary cancer. Based on these observations, we conclude that in mammary cancer, VEGF can regulate, in addition to endothelial proliferation and angiogenesis, also growth of tumour cells by an autocrine mechanism mediated through its receptors.

### MATERIALS AND METHODS

### Animals and treatment

All animals used in this study were 3-month-old, sexually mature Noble rats weighing 180–210 g at the beginning of the experiment. These rats were surgically implanted subcutaneously (s.c.) in the inguinal region, under pentobarbitone anaesthesia, with four 2.0-cm long Silastic tubings (inner diameter 1.6 mm, outer diameter 3.2 mm) containing testosterone propionate (T) and one 1.0-cm long Silastic tubing containing 17β-oestradiol ($E_2$). The rats were palpated regularly for mammary tumours starting from 2 months after treatment. The rats were killed when moribund or when a tumour > 2 cm in diameter were detected. All rats used were bred in the Department and had tap water and diet available ad libitum at all times.

### Histopathological examination

The mammary tumours were fixed immediately after removal in 10% formalin buffered with 0.1 M phosphate buffer (pH 7.2), trimmed and embedded in paraffin. Paraffin sections (4-μm in thickness) were prepared and stained with haematoxylin and eosin (H&E) for histopathological examination, using the criteria and classification of mammary tumours as outlined by Russo et al (1990).

### Single-labelling immunohistochemistry

Immunohistochemical staining for VEGF was performed on formalin-fixed paraffin sections, using an avidin–biotin immunoperoxidase technique. A rabbit polyclonal and a goat polyclonal antibodies for rat VEGF (Santa Cruz Biotechnology, Santa Cruz, CA, USA) were used at a 1:50 and 1:100 dilutions respectively. The sections were dewaxed in xylene and rehydrated through graded alcohol before being incubated for 20 min in 0.3% hydrogen peroxide (v/v) in methanol to inhibit endogenous peroxidase. The antibody binding epitope of the antigen was retrieved by microwave treatment for 10 min in boiling 10 mM citrate buffer (pH 6.0). The slides were allowed to cool for 20 min in the citrate buffer before further treatment. After a quick rinse in distilled water and in phosphate-buffered saline (PBS), the sections were treated with blocking solution from EliteABC-kit (Vector, Peterborough, UK) for 20 min. After blotting off the blocking solution, the sections were covered with primary antiserum against VEGF and incubated for 1 h at 37°C in a humid chamber. The sections were then incubated with the appropriate biotinylated secondary antibody at 1:100 dilution followed by peroxidase-conjugated avidin–biotin complexes according to the manufacturer's instructions (EliteABC-kit, Vector, Peterborough, UK, and diaminobenzidine (DAB). The sections were then counterstained with Meyer's haematoxylin.

Immunocytochemical staining with rabbit polyclonal antibody to flt-1 and flk-1 (Santa Cruz Biotechnology, Santa Cruz, CA, USA) was performed in the same way as that of VEGF, except that microwave treatment was omitted. The dilutions of antibodies for flt-1 and flk-1 were 1:100 and 1:50 respectively.

### Double-labelling immunocytochemistry

To determine the relationship between cell proliferation and expression of VEGF, flt-1, or flk-1, double immunocytochemical studies for both Ki-67 and VEGF, flt-1, or flk-1 were performed. Immunocytochemical staining for VEGF, flt-1 and flk-1 was performed as described above. The sections were then treated for 10 min in boiling 10 mM citrate buffer (pH 6.0) in a microwave oven to block antibody cross-reactivity and to retrieve antigens. A mouse monoclonal antibody against Ki-67 (Novocastra, Newcastle upon Tyne, UK) was used at 1:500 dilution. The sections were incubated with the appropriate biotinylated secondary antibody (1:100) followed by alkaline phosphatase conjugated avidin–biotin complexes according to the manufacturer's instructions (ABC-AP kit, Vector, Peterborough, UK). Ki-67 reactivity was recognized as diffuse or dot-like red nuclear.

### Controls for immunocytochemistry

The specificity of the reactivity of each antibody was checked by the following controls: (a) normal adult rat brain as a negative control for all antibodies (Plate et al, 1993, 1994); (b) fetal rat kidney sections were used as positive control for antibody against VEGF, flt-1 and flk-1 (Shim et al, 1996); (c) fetal rat kidney and skin serving as positive controls for antibody against Ki-67; (d) preabsorption of the antisera against VEGF, flt-1 and flk-1 with 50-fold concentration of VEGF peptide, flt-1 peptide and flk-1 peptide respectively; (e) preabsorption of antisera with non-specific antigens such as epidermal growth factor (EGF) and basic fibroblast growth factor (bFGF); (f) replacement of specific primary antibodies with the corresponding normal serum.

### Evaluation of immunoreactivities of VEGF and its receptors and Ki-67 labelling index

The intensity of immunostaining for VEGF, flt-1 and flk-1 was scored as weak and strong. The Ki-67-positive cells were evaluated by ten high-power fields selected according to the intensities of immunoreactivity for VEGF, flt-1 and flk-1, in six specimens. Results were given as mean and standard deviation (s.d.). The Kruskal–Wallis test was applied to correlate the Ki-67 labelling index (LI) to the intensity of staining for VEGF, flt-1 and flk-1 (Dellas et al, 1996). A value of $P < 0.05$ was considered to be statistically significant.

### Western blot analysis

Protein extracts were prepared by homogenizing thawed tissue in a modified radioimmunoprecipitation buffer (50 mM Tris–HCl,

 © 1999 Cancer Research Campaign

150 mM sodium chloride, 1 mM EDTA, 1% NP-40, 0.5% sodium deoxycholate, 0.1% sodium dodecyl sulphate (SDS)) containing a cocktail of protease and phosphatase inhibitors (2 mM phenylmethylsulphonyl fluoride, 10 µg $ml^{-1}$ each of aprotinin, leupeptin and pepstatin A, 1 mM $Na_3VO_4$, 50 mM NaF, 5 mM sodium pyrophosphate), using a tissue tearor (Biospec Products, Inc.). The tissue lysate was centrifuged at 14 000 rpm for 30 min at 4°C. The supernatant was collected and its protein content was measured using DC protein assay (Bio-Rad, Hercules, CA, USA). Equal amounts of protein (100 µg) were separated by SDS polyacrlamide gel electrophoresis (SDS-PAGE). The electrofractionated proteins were transferred to polyvinyldifluoride (PVDF) membranes (Bio-Rad, Hercules, CA, USA). Blocking and washing steps were performed according to the ECL instruction manual (Amersham Pharmacia Biotech UK Ltd, Buckinghamshire, UK). The blots were probed for 1–2 h with primary antibodies (anti-flt-1 or anti-flk-1 (Santa Cruz Biotechnology, Inc., Santa Cruz, CA, USA)), which were the same as those used in the immunohistochemistry, followed by incubation with anti-rabbit IgG conjugated to horseradish peroxidase (Amersham Pharmacia Biotech UK Ltd, Buckinghamshire, UK). The immunoreactive signals were detected by ECL Western blot detection reagents (Amersham Pharmacia Biotech UK Ltd, Buckinghamshire, UK) following the manufacturer's instructions. For controls, the antibodies were preincubated with ten fold excess (by weight) of specific corresponding blocking peptides (flt-1 or flk-1 (Santa Cruz Biotechnology, Inc., Santa Cruz, CA, USA)) for 2 h at room temperature. This competition procedure was used to validate the specificity of each antibody reagent used for immunoblots.

### RESULTS

### Hormone-induced mammary cancer

Mammary carcinoma can be induced readily by the combination of testosterone and oestradiol protocols as outlined. Fully developed mammary cancers can be consistently induced between 4 and 6 months. Most tumours observed here were ductal carcinoma of moderately differentiated to poorly differentiated type. The detailed histopathology of this induced cancer is described elsewhere (Wong et al, 1999; Xie et al, 1999*a*, 1999*b*, 1999*c*). Many glands had tumour cells completely filling the lumen of mammary ducts (carcinoma in situ, Figure 1), while others had obviously broken out from the ductal structure and invaded the stromal tissues (invasive carcinoma, Figure 2). Isolated clumps or trabeculae of tumour cells were observed frequently interspersed by stromal connective tissue which was often infiltrated with lymphocytic cells. The cancer cells were typically pleomorphic with highly variable nuclear size and chromatin density.

### Expression of VEGF

The expression of VEGF in mammary ductal carcinoma in situ (Figure 3) as well as in invasive mammary cancer (Figure 4) were examined. Most tumour cells were moderately or strongly stained by the anti-VEGF antibody, suggesting the presence of VEGF protein in these cells. Adjacent, morphologically 'normal' ductal structures were negative to VEGF (Figure 3). On the other hand, tumour cells that had invaded the stromal connective tissue (Figure 4) were more strongly stained by the antibody than those in mammary cancer in situ. In control specimens all of them reacted negatively to normal serum and the antibody preabsorbed with VEGF peptide, indicating that the staining was specific for VEGF (Figure 9).

**Table 1** Correlation between expression of VEGF and its receptors and Ki-67 index

| | Ki-67 index % (mean ± s.e.m.) | | |
|---|---|---|---|
| | Weak | Strong | *P*-value |
| VEGF expression | 3.7 ± 0.9 | 58.3 ± 8.6 | <0.001 |
| Flk-1 expression | 3.1 ± 1.1 | 53.9 ± 9.2 | <0.001 |
| Flt-1 expression | 34.6 ± 9.1 | 18.4 ± 2.7 | <0.001 |

### Expression of VEGF receptors

The results showed that both flk-1 and flt-1 were present, in addition to endothelium, in cancer cells (Figures 5, 6, 7, 8). Closer examination showed that there was a difference in distribution between the two receptors, flk-1 and flt-1. The former was present generally in most cancer cells irrespective of whether they were in situ or invasive (Figures 5, 6). However, for flt-1, the invasive tumour was much more strongly expressed than the in situ form of cancers (Figures 7, 8). Interestingly, in one duct (Figure 5, arrowhead), in which part of it was occupied by malignant cells while the remaining was lined by normal cells, the malignant cells all expressed flk-1, but not the normal cells. Both flt-1 and flk-1 were negative in adjacent normal ductal epithelial cells (Figures 5, 7). Preincubation with flk-1 peptide yielded a negative result (Figure 10).

### Correlation between expression of VEGF and Ki-67 index

In order to establish the relationship between VEGF and Ki-67 index, a double-labelling immunocytochemical method was employed. The results showed that the intensity of VEGF staining was heterogeneous within the cancer tissue. In an attempt to understand better the significance of heterogeneity in VEGF expression within the tumour, we assessed the reactivities of this factor against Ki-67 signal. Of the 1000 cells examined in the area of strong VEGF reactivity, 58.3% of cells were also Ki-67-positive. This was much higher than in the weak VEGF reactive area ($P < 0.001$; Table 1). This means that the reactivity of VEGF in tumour cells appeared to be positively correlated with the Ki-67 overexpression. A good example is shown in Figures 11 and 12, where a large number of Ki-67-positive cells are seen in strong VEGF reactive zone. By contrast, the weak VEGF reactive zone in the mammary carcinoma has a lower Ki-67 index. Taken together, these results indicated that the expression of VEGF is closely correlated to the proliferation of mammary cancer cells.

### Correlation between expression of VEGF receptors and Ki-67 index

To determine which receptor, flt-1 or flk-1 or both, mediated proliferation signals, we further examined the correlation between expression of VEGF receptors and Ki-67 index. Based on the

© 1999 Cancer Research Campaign 

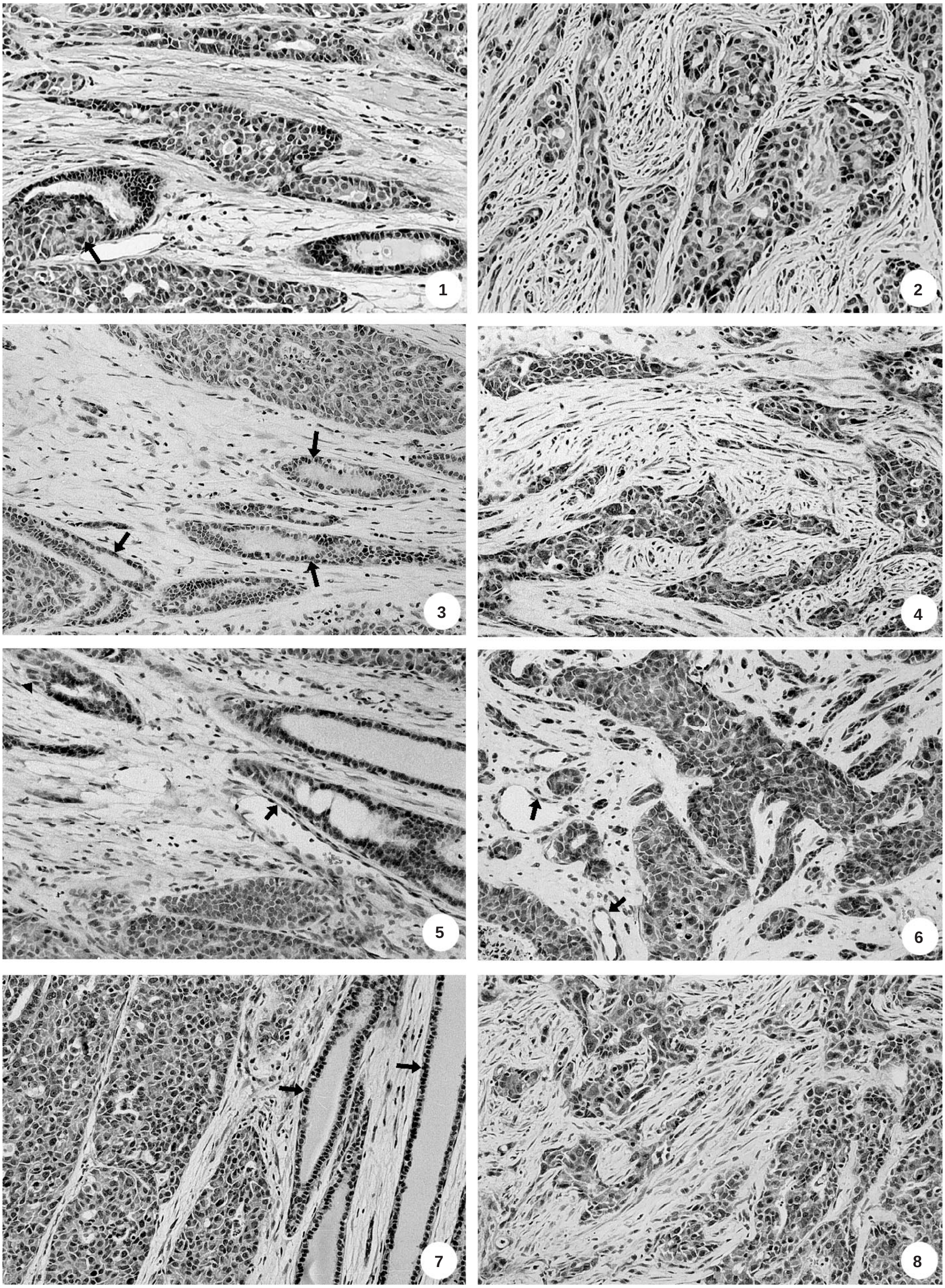

**Figure 1** Mammary cancer in situ. The tumour cells completely fill the lumen of mammary ducts. An intraductal carcinoma (arrow), illustrating possibly the evolution of malignancy from a dysplastic site. 150×. **Figure 2** Invasive mammary cancer. The tumour cells have obviously broken out from the ductal structural confine and invaded the stromal tissues. Isolated clumps or trabeculae of tumour cells are seen interspersed within the stromal connective tissue which is often infiltrated with lymphocytic cells. 150×. **Figures 3 and 4** Expression of VEGF in mammary cancer in situ (**Figure 3**) and in invasive mammary cancer (**Figure 4**). Most tumour cells are moderately or strongly stained by the anti-VEGF antibody. The invasive tumour cells are more strongly stained by this antibody. Note that the adjacent morphologically normal ductal structures are negative to VEGF (arrows). This indicates that VEGF overexpression occurs in tumour cells only. 150×. **Figures 5 and 6** Expression of flk-1 in mammary cancer in situ (**Figure 5**) and in invasive mammary cancer (**Figure 6**). The flk-1 reactivity is seen not only in endothelial cells (arrows) but also generally in most tumour cells, irrespective of whether they are in situ or invasive. The ductal cells, indicated by an arrowhead are positive, despite the general negativity of the duct. 150×. **Figures 7 and 8** Expression of flt-1 in mammary cancer in situ (**Figure 7**) and in invasive mammary cancer (**Figure 8**). The flt-1-positive staining cells are seen scattered in mammary cancer. The intensity of flt-1 staining is more strongly expressed in invasive than in situ mammary cancer. Note negative reaction in normal ducts in Figure 7 (arrows). 150×. Legends are based on original colour micrographs submitted for review.

 *© 1999 Cancer Research Campaign*

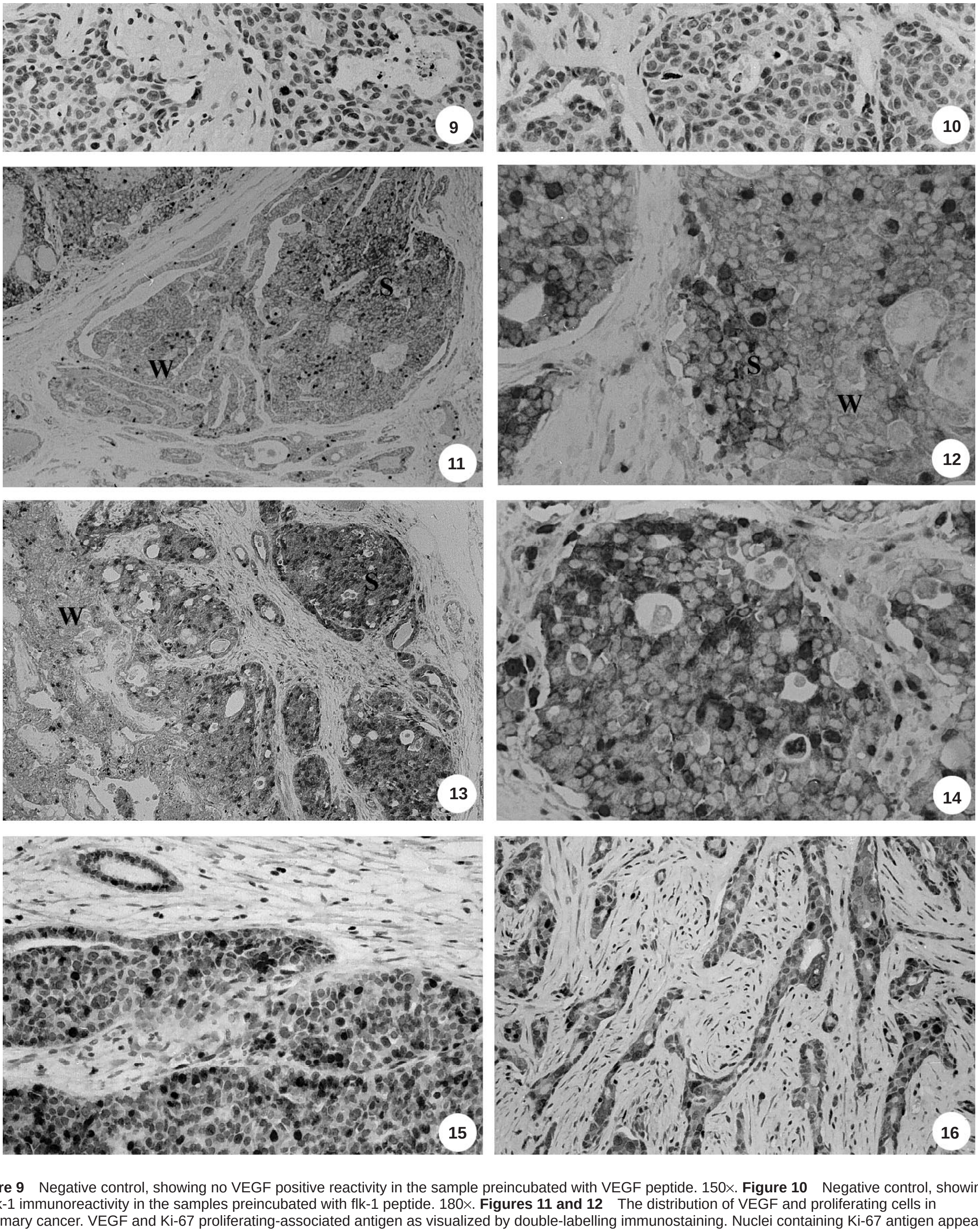

**Figure 9** Negative control, showing no VEGF positive reactivity in the sample preincubated with VEGF peptide. 150×. **Figure 10** Negative control, showing no flk-1 immunoreactivity in the samples preincubated with flk-1 peptide. 180×. **Figures 11 and 12** The distribution of VEGF and proliferating cells in mammary cancer. VEGF and Ki-67 proliferating-associated antigen as visualized by double-labelling immunostaining. Nuclei containing Ki-67 antigen appear red, whereas cytoplasm containing VEGF appear brown. The strong (S) and weak (W) areas of VEGF reactivities are most obvious at this low magnification as illustrated in Figure 11. Figure 12 is a higher magnification of a VEGF site with Ki-67-positive cells. Note the positive correlation of Ki-67 to reactivity of VEGF. 60× (**Figure 11**), 320× (**Figure 12**). **Figures 13 and 14** The distribution of flk-1 and proliferating cells in mammary cancer. Flk-1 and Ki-67 proliferating-associated antigen as visualized by double-labelling immunostaining. Nuclei containing Ki-67 antigen appear red, whereas flk-1 positive cells appear brown in colour. The strong (s) flk-1 site as illustrated in (**Figure 13**) is shown at higher power in **Figure 14**. Note the positive correlation of Ki-67 to flk-1 immunoreactivity. 60× **Figure 13**, 320× (**Figure 14**). **Figures 15 and 16** The distribution of flt-1 and proliferating cells in mammary cancer in situ (**Figure 15**) and invasive mammary cancer (**Figure 16**). The correlation between flt-1 positivity and Ki-67 is not easily apparent. The Ki-67-positive cells appear to scatter more randomly within the tumour. However, morphometric measurement shows that flt-1 activity is inversely related to Ki-67 activity; i.e. a lower flt-1 is correlated with higher Ki-67 reactivity. 150×. Legends are based on original colour micrographs submitted for review.

*© 1999 Cancer Research Campaign*

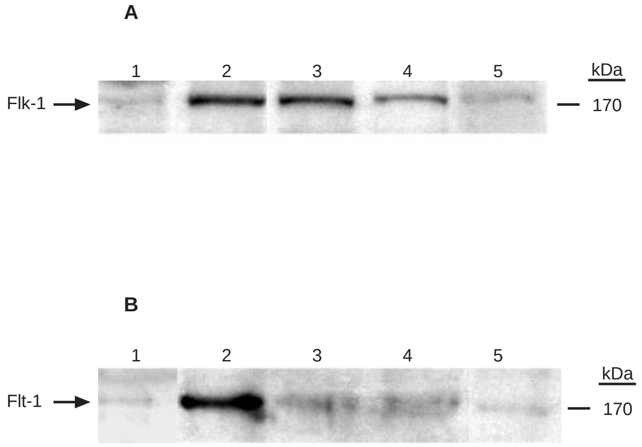

**Figure 17** Western blot analyses of flk-1 (**A**) and flt-1 (**B**). Proteins were extracted from normal mammary gland tissue from untreated control rats and mammary tumours induced by combination of testosterone and 17β-oestradiol ($1E_2$+4T). Equal amounts of proteins (100 μg $lane^{-1}$) were loaded into the gel and membranes were probed with the rabbit anti-flk-1 antibody (**A**) or the rabbit anti-flt-1 antibody (**B**). The molecular weight standards, as indicated on the right in each blot are 170 kDa (**A** and **B**). The lanes 1, in both **A** and **B**, represent extracts from normal mammary gland tissues. The lanes 2–4 are extracts from individual mammary tumours, and the lanes 5 in both **A** and **B** are the tumour extracts from lanes 2 in **A** and **B** respectively, in which the primary antibodies, anti-flk-1 or anti-flt-1, were preincubated with their respective blocking peptides. In **A**, the flk-1 protein (~170–175 kDa) is highly expressed in mammary tumours (lanes 2–4) but is very weakly expressed in normal mammary gland (lane 1). This band is abolished in the blot preincubated with anti-flk-1 antibody and flk-1 peptide (lane 5). In **B**, the overall expression of flt-1 protein (~165–170 kDa) in mammary tumours (lanes 2–4) is high relative to the normal control (lane 1) but its expression varied among different tumour samples. This band is attenuated by preincubation with anti-flt-1 antibody and flt-1 peptide reactivities of the tumour to antiserum for flk-1, a similar pattern of strong and weak receptor reactive zones were established. When these sections were treated with Ki-67 antiserum, most of the Ki-67-positive cells were found to be located in the strong receptor reactive zone. Ki-67 indices were found to be 53.9% and 3.1% in the strong and weak reactive zones for flk-1 respectively (Table 1; Figures 13 and 14). On the other hand, the flt-1-positive cells were often scattered within cancer tissues. Of the 1000 cells examined from strong reactive area, 18.5% expressed immunodetectable Ki-67. By contrast, the Ki-67 index for low flt-1 reactive areas was 34.6%. Figures 15 and 16 show the non-segregation pattern between proliferating cells (Ki-67-positive cells) and those expressing flt-1 in mammary cancer in situ (Figure 15) and invasive mammary cancer (Figure 16). These results suggested that, unlike flk-1, flt-1 reactivity of tumour cells appeared to be inversely correlated to proliferation marker of this tumour. These results suggested that flk-1 rather than flt-1 was responsible for VEGF autocrine proliferative regulation in mammary cancer. It was also noted that the invasive tumour, though having a high flt-1 reactivity, had a lower Ki-67 index.

### Western blot analysis

Immunoblots of tissue extracts from samples of mammary tumour (lanes 2–4 in Figure 17A) when probed with the rabbit anti flk-1 antibody revealed a strong single band of approximately 170–175 kDa that was only weakly expressed in the normal mammary glands (lane 1 in Figure 17A). Preincubation of the anti-flk-1 antibody with the corresponding flk-1 peptide abolished the 170–175 kDa band (lane 5 in Figure 17A), demonstrating the specificity of this antibody. On the other hand, a distant band with an apparent molecular mass of 165–170 kDa was observed in immunoblots of extracts of mammary tumours (lanes 2–4 in Figure 17B) when probed with the rabbit anti-flt-1 antibody. However, the 165– 170 kDa band exhibited variable levels of expression among different tumour samples. In normal mammary glands, the expression of this band was consistently low (lane 1 in Figure 17B). Preblot incubation of the flt-1 peptide with the antibody results in significant reduction in the 165–170 kDa band (lane 5 in Figure 17B), showing the specificity of this immunoblot analysis.

The flk-1 band (~ 170–175 kDa) and the flt-1 band (~ 165–170 kDa) recognized in our immunoblot studies shows a very close agreement with molecular masses reported for the rat flk-1 (about 180 kDa) and rat flt-1 (about 180 kDa) (Yamane et al, 1994). In retina, Yang and Cepko (1996) observed that chick flk-1 protein showed a slightly higher molecular weight (~200 kDa) than murine flk-1 protein (~180 kDa). Takahashi and Shibuya (1997) observed that an immature form of human flk-1 with a molecular mass of about 150 kDa could be glycosylated to create a 200 kDa intermediate, and after further glycosylation a mature 230 kDa was expressed on the cell surface. Thus, the minor difference in molecular weights we observed may be attributed to the difference of animal species and/or the differential status in post-translational glycosylations (Seetharam et al, 1995). Our immunoblot studies showed that both flk-1 and flt-1 are overexpressed in mammary tumours but the level of expression of flt-1 is variable among different tumour cases, and confirmed the specificity of the anti-flk-1 and the anti-flt-1 antibodies used in our immunohistochemistry.

### DISCUSSION

We have demonstrated that, in an animal model developed in our laboratory, the co-expression of VEGF and its receptors, flt-1 and flk-1, in mammary cancer cells in vivo. Not only have we found that mammary tumour cells expressed high levels of VEGF, which is consistent with previous observations (Hendrix et al, 1997), but also, surprisingly, the expression of its receptors, flt-1 and flk-1, in mammary cancer cells. Intense reactivities to VEGF, flt-1, and flk-1 were observed in tumour cells, especially in invasive tumour lesions. Furthermore, the intensity of immunostaining for VEGF was very closely correlated with that of flk-1. Similar finding was also reported by Takahashi et al (1995). They observed that most tumours with high levels of VEGF were associated with flk-1 positivity in their endothelial cells, indicating that VEGF can upregulate its own receptor.

An important question to consider was whether the immunoreactivities for all antibodies were specific, or whether the positive reactivities for the antibodies were false positive. To answer this question, adult rat brain tissue was chosen as the negative control for all antibodies as it is well established that adult brain does not have detectable levels of VEGF and its receptors. Negative reactivity was observed in all negative control sections, whereas the positive reactivities for VEGF, flt-1 and flk-1 were observed in fetal rat kidney, a tissue known to contain VEGF. On the other hand, preincubation of antisera with 50-fold excess of the corresponding antigens (in concentration) all gave a negative result in

 © 1999 Cancer Research Campaign

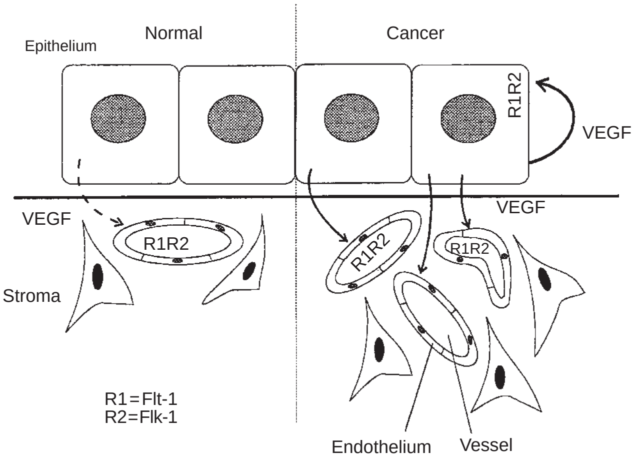

**Figure 18** Schematic representation of VEGF and its receptors in mammary cancer. In normal mammary gland epithelial cells secrete very small, undetectable amount of VEGF to maintain the growth of blood vessels (indicated by dotted line arrow). In mammary carcinoma there is not only an up-regulation of VEGF in cancer cells to induce angiogenesis but also an overexpression of VEGF receptors, flt-1 (R1) and flk-1 (R2). This indicates that there is an autocrine signalling pathway for regulating the growth and metastasis in mammary cancer cells. The thin arrows indicate a paracrine signalling pathway for angiogenesis, whereas the thick arrow indicates the autocrine loop for growth and metastatic regulation in mammary cancer cells addition to omission of primary antisera. These results provided further evidence for the specificity of the immunostaining in our study.

Western blot experiments demonstrated an increase in flk-1 protein expression in induced mammary cancers. The molecular size of flk-1 is about 170–175 kDa, which is consistent with other researcher's reports (Yamane et al, 1994; Seetharam et al, 1995; Yang and Cepko, 1996; Takahashi and Shibuya, 1997). Angiogenesis is essential for development of tumours, including breast cancer, because angiogenesis supplies not only nutrients (especially the paracrine stimulators, such as numerous growth factors, for tumour cells) and oxygen requirements for the growing tumour but also provide vascular route for metastasis. Therefore, angiogenesis is a significant prognostic indicator for breast cancer (Hanahan et al, 1996). Among all factors, VEGF is one of the most potent inducer of angiogenesis (Hanahan et al, 1996). It is substantially overexpressed in many transformed cell lines and in malignant tumours (Gitay-Goren et al, 1993; Shibuya, 1995; Enomoto et al, 1994), which are closely correlated with the poor prognosis of many malignant tumours (Tio et al, 1994). To date, VEGF is known to be a specific growth factor for endothelial cells because it has been reported that VEGF receptors, flt-1 and flk-1, are exclusively present in endothelium (Dvorak et al, 1995; Ferrara, 1995). Although some studies have indicated that receptors are also expressed by several non-endothelial cells, the binding of VEGF to the receptors on these cells does not seem to induce cell proliferation. Rather, it has been noted that VEGF induces motility of monocytes, differentiation of osteoblasts, production of insulin by beta cells, and disorganization of actin stress fibres in Balb/c 3T3 cells (Enomoto et al, 1994). These suggest that VEGF is a multifunctional growth factor in different type of cells. On the other hand, some researchers have observed the correlation between expression of VEGF and proliferation of tumour cells. However, they ascribe the increase of proliferative index to the angiogenic activity of VEGF because the receptors for VEGF are restricted to endothelial cells rather than on tumour cells (Takahashi et al, 1995). Taken together, most studies to date have been concerned with the effects of VEGF on angiogenic activity.

The observation of receptors in the mammary cancer cells which overexpress VEGF led us to hypothesize that a VEGF autocrine loop may exist in mammary cancer (as illustrated in Figure 18). To verify this hypothesis, we employed a double-labelling immunostaining approach to determine whether proliferation of mammary cancer cells was correlated with the expression of VEGF, flt-1 and flk-1. We resorted to using Ki-67 as a proliferation marker because Ki-67 was considered to be the best for use on conventional sections (Rose et al, 1994). Using Ki-67 LI as a parameter we evaluated the proliferation of mammary cancer cells. Unlike the relative long half-life of proliferating cell nuclear antigen (PCNA), which affects the accuracy of scoring, the Ki-67 protein has very short half-life of only approximately 20 min within the cycle. It is almost totally catabolized at the completion of mitosis, thus, only very few cells that have left the cycle would contain this antigen (Ross et al, 1995). Ki-67 has thus been applied to investigate the growth fractions and cytokinetic activities in mammary cancer. It has been shown that Ki-67 gives a rapid and reliable estimate of the neoplastic cell growth fractions (Gerdes et al, 1986). Moreover, in a retrospective study, Wintzer et al (1991) have reported that the growth fraction measured by Ki-67 was found to correlate directly with the T stage, nodal status and tumour grading. In our study, Ki-67 LI was significantly higher in cells with strong reactivities to VEGF than those with weak reactivities. This indicates a correlation between expression of Ki-67 and VEGF. This means that tumour cells that express high VEGF have a higher proliferation rate. Similar correlation was also observed between Ki-67 and flk-1, but not with flt-1. These data provide evidence to support our hypothesis that VEGF may be an autocrine growth factor for mammary cancer cells and that the autocrine growth regulatory role may be mediated through flk-1.

Although flt-1 is also known to be a receptor for VEGF, recent experiments have demonstrated that flt-1 do not mediate a classical proliferative signal (Waltenberger et al, 1994). Our results support these observations since the expression of flt-1 seems to be inversely correlated with high Ki-67 LI. Fong et al (1995) have reported that targeted deletion of the flt-1 gene resulted in abnormal blood vessel development and early embryonic lethality. They pointed out that VEGF binding to flt-1 elicits endothelial cell–cell interactions and capillary tube formation, a process that follows closely the proliferation and migration of endothelial cells. Others have reported that flt-1 may play a role in the process of endothelial reorganization (Millauer et al, 1993). These observations suggested that flt-1 may be related to the migration of endothelial cells. In our study, we have observed that flt-1 was expressed mainly in invasive mammary cancer cells and that flt-1 is inversely correlated to Ki-67 index. These results lead us to speculate that expression of flt-1 may be responsible for the invasive growth and metastasis of mammary cancer. Western blot analysis revealed an increase in flt-1 protein expression in all, though at variable levels in mammary cancers and the molecular size of flt-1 is about 170 kDa, which is consistent with other researcher's observations (Mitola et al, 1997). This result may provide preliminary evidence to support our speculation. However, the precise role of flt-1 in mammary cancer requires further study.

Furthermore, we have established a cell line from the animal model and observed that this cell line expressed high levels of VEGF and its receptors (Xie et al, 1999*d*). In order to further

© 1999 Cancer Research Campaign 

confirm the autocrine growth stimulatory action of VEGF in mammary cancer cells, we treated the cells with exogenous synthetic VEGF and observed an apparently increased growth rate in the treated cells (data not shown). On the other hand, we have applied the same antibodies to ten human mammary cancer samples. The results were same as observed in our animal model (data not shown). Taken together, the in vitro study and investigation on human tissues provide further evidence to support our hypothesize that VEGF may act as an autocrine growth regulator.

In summary, in this report we have demonstrated that (i) mammary cancer cells express high level of VEGF as well as its receptors (flt-1 and flk-1); (ii) expression of VEGF is quantitatively related to proliferation of mammary cancer cells; and (iii) the proliferation of tumour cells is quantitatively correlated to the expression of flk-1, but not to flt-1. Our findings reveal that VEGF may act as an autocrine growth factor for mammary cancer cells in vivo and this autocrine regulatory role may be mediated by flk-1. The current study has, therefore, shed a new light on the mechanism of mammary carcinogenesis.

### REFERENCES

Dellas A, Schultheiss E, Oberholzer M, Torhorst J and Gudat F (1996) Analysis of proliferative activity using Ki-67 on cervical precancerous lesions and the relationship to p53 expression. *Anticancer Res* **16**: 3403–3408

Dvorak H, Brown L, Detmar M and Dvorak A (1995) Vascular permeability factor/vascular endothelial growth factor, microvascular hyperpermeability, and angiogenesis. *Am J Pathol* **146**: 1029–1039

Enomoto T, Okamoto T and Sato JD (1994) Vascular endothelial growth factor induces the disorganization of actin stress fibers accompanied by protein tyrosine phosphorylation and morphological change in Balb/C3T3 cells. *Biochem Biophys Res Commun* **202**: 1716–1723

Ferrara N (1995) The role of vascular endothelial growth factor in pathological angiogenesis. *Breast Cancer Res Treat* **36**: 127–137

Ferrara N, Houck KA, Jakeman LB and Leung DW (1991) The vascular endothelial growth factor family of polypeptides. *J Cell Biochem* **47**: 211–218

Fong GH, Rossant J, Gertsenstein M and Breitman ML (1995) Role of the Flt-1 receptor tyrosine kinase in regulating the assembly of vascular endothelium. *Nature* **376**: 66–70

Fox SB and Harris AL (1997) Markers of tumor angiogenesis: clinical applications in prognosis and anti-angiogenic therapy. *Invest New Drugs* **15**: 15–28

Gasparini G, Toi M, Gion M, Verderio P, Dittadi R, Hanatani M, Matsubara I, Vinante O, Bonoldi E, Boracchi P, Gatti C, Suzuki H and Tominaga T (1997) Prognostic significance of vascular endothelial growth factor protein in node-negative breast carcinoma. *J Natl Cancer Inst* **89**: 139–147

Gerdes J, Lelle RJ and Pickartz H (1986) Growth fractions in breast cancer determined in situ with the monoclonal antibody Ki-67. *J Clin Pathol* **39**: 977–980

Gitay-Goren H, Halaban R and Neufeld G (1993) Human melanoma cells but not normal melanocytes express vascular endothelial growth factor receptors. *Biochem Biophys Res Commun* **190**: 702–708

Hanahan D (1997) Signaling vascular morphogenesis and maintenance. *Science* **277**: 48–50

Hanahan D and Folkman J (1996) Patterns and emerging mechanisms of the angiogenic switch during tumorigenesis. *Cell* **86**: 353–364

Hendrix MJC, Muschel RJ and Padarathsingh M (1997) Recent advances in breast cancer research: from genes to management. *Am J Pathol* **151**: 883–888

Martin L, Green B, Renshaw C, Lowe D, Rudland P, Leinster SJ and Winstanley L (1997) Examining the technique of angiogenesis assessment in invasive breast cancer. *Br J Cancer* **76**: 1046–1054

Masood R, Cai J, Zheng T, Smith DL, Naidu Y and Gill PS (1997) Vascular endothelial growth factor/vascular permeability factor in an autocrine growth factor for AIDS-Kaposi sarcoma. *Proc Natl Acad Sci USA* **94**: 979–984

Millauer B, Wizigmann-Voos S, Schnurch H, Martinez R, Moller NPH, Risau W and Ullrich A (1993) High affinity VEGF binding and developmental expression suggest Flk-1 as a major regulator of vasculogenesis and angiogenesis. *Cell* **72**: 835–846

Mitola S, Sozzani S, Luini W, Primo L, Borsatti A, Weich H and Bussolino F (1997) Tat-human immunodeficiency virus-1 induces human monocyte chemotaxis by activation of vascular endothelial growth factor receptor-1. *Blood* **90**: 1365–1372

Parker SL, Tong T, Bolden S and Wingo PA (1997) Cancer statistics 1997. *CA Cancer J Clin* **47**: 5–27

Plate KH, Breier G, Millauer B, Ullrich A and Risau W (1993) Up-regulation of vascular endothelial growth factor and its cognate receptors in a rat glioma model of tumor angiogenesis. *Cancer Res* **53**: 5822–5827

Plate KH, Breier G, Weich HA, Mennel HD and Risau W (1994) Vascular endothelial growth factor and glioma angiogenesis: coordinate induction of VEGF receptors, distribution of VEGF protein and possible in vivo regulatory mechanisms. *Int J Cancer* **59**: 520–529

Ref M, Lejeune S, Scott PA, Fox S, Smith K, Leek R, Moghaddam A, Whitehouse R, Bicknell R and Harris AL (1997) Expression of the angiogenic factors vascular endothelial cell growth factor, acidic and basic fibroblast growth factor, tumor growth factor beta-1, platelet-derived endothelial cell growth factor, placenta growth factor, and pleiotrophin in human primary breast cancer and its relation to angiogenesis. *Cancer Res* **57**: 963–969

Rose DSC, Maddox PH and Brown DC (1994) Which proliferation markers for routine immunohistology? A comparison of five antibodies. *J Clin Pathol* **47**: 1010–1014

Ross W and Hall PA (1995) Ki-67: from antibody to molecule to understanding. *J Clin Pathol:Clin Mol Pathol* **218**: M113–M117

Russo J, Russo IH, Rogers AE, Van Zwietan MJ and Gusterson B (1990) Tumors of the mammary gland. *IARC Scientific Publications* **99**: 47–78

Seetharam L, Gotoh N, Maru Y, Neufeld G, Yamaguchi S and Shibuya M (1995) A unique signal transduction from FLT tyrosine kinase, a receptor for vascular endothelial growth factor VEGF. *Oncogene* **10**: 135–147

Senger DR, Galli SJ, Dvorak AM, Perruzzi CA, Harvey VS and Dvorak HF (1983) Tumor cells secrete a vascular permeability factor that promotes accumulation of ascites fluid. *Science (Washington DC)* **219**: 983–985

Senger DR, Van De Water L, Brown L, Nagy J, Yeo K-T, Yeo T-K, Berse B, Jackman R, Dvorak A and Dvorak H (1993) Vascular permeability factor (VPF, VEGF) in tumor biology. *Cancer Metastasis Rev* **12**: 303–324

Shibuya M (1995) Role of VEGF-Flt receptor system in noral and tumor angiogenesis. *Adv Cancer Res* **67**: 281–316

Shim JW, Koh YC, Ahn HK, Park YE, Hwang DY and Chi JG (1996) Expression of bFGF and VEGF in brain astrocytoma. *Journal of Korean Medical Science* **11**: 149–157

Takahashi T and Shibuya M (1997) The 230 kDa mature form of KDR/Flk-1 (VEGF receptor-2) activates the PLC-gamma pathway and partially induces mitotic signals in NIH3T3 fibroblasts. *Oncogene*, **14**: 2079–2089

Takahashi Y, Kitadai Y, Bucana CD, Cleary KR and Ellis LM (1995) Expression of vascular endothelial growth factor and its receptor, KDR, correlates with vascularity, metastasis, and proliferation of human colon cancer. *Cancer Res* **55**: 3964–3968

Tio M, Hoshina S, Takayanagi T and Tominaga T (1994) Association of vascular endothelial growth factor expression with tumor angiogenesis and with early relapse in primary breast cancer. *Jpn J Cancer Res* **85**: 1045–1049

Waltenberger J, Claesso-Welsh L, Siegbahn A, Shibuya M and Heldin CH (1994) Different signal transduction properties of KDR and FLT1, two receptors for vascular endothelial growth factor. *J Biol Chem* **269**: 26988–26995

Wintzer H-O, Schulte-Monting J, Hellerich U and Von Kleist S (1991) Ki-67 immunostaining in human breast tumors and its relationship to prognosis. *Cancer* **67**: 421–428

Wong YC, Xie B and Tsao GSW (1999) Induction of breast cancer in Noble rats with a combination of estrogen and testosterone. In: *Hormonal Carcinogenesis III*, Li JJ, Daling J and Li SA (eds). Springer-Verlag. New York

Xie B, Tsao SW and Wong YC (1999*a*) Induction of high incidence of mammary tumor in female Noble rats with a combination of 17β-oestradiol and testosterone. *Carcinogenesis*, **20**: (in press)

Xie B, Tsao SW and Wong YC (1999*b*) Sex hormone-induced mammary carcinogenesis in the female Noble rats: the role of androgens. *Carcinogenesis* **20**: 1069–1078

Xie B, Tsao SW and Wong YC (1999*c*) Sex hormone-induced mammary carcinogenesis in the female Noble rats: expression of TGF-$\beta_1$ and its receptors, TGF-α, and EGF-R in mammary carcinogenesis. *Breast Cancer Res Treat* 1597–1606

Xie B, Tsao SW and Wong YC (1999*d*) Isolation and purification of breast carcinoma cell lines from a Noble rat. In: *Hormonal Carcinogenesis III*, Li JJ, Daling J and Li SA (eds) Springer-Verlag; New York

Yamane A, Seetharam L, Yamaguchi S, Gotoh N, Takahashi T, Neufeld G and Shibuya M (1994) A new communication system between hepatocytes and

 © 1999 Cancer Research Campaign sinusoidal endothelial cells in liver through vascular endothelial growth factor and Flt tyrosine kinase receptor family (Flt-1 and KDR/Flk-1). *Oncogene* **9**: 2683–2690

Yang X and Cepko CL (1996) Flk-1, a receptor for vascular endothelial growth factor (VEGF) is expressed by retinal progenitor cells. *J Neurosci* **16**: 6089–6099

© 1999 Cancer Research Campaign 
